# Pleural Infection: Diagnosis, Management, and Future Directions

**DOI:** 10.3390/jcm14051685

**Published:** 2025-03-02

**Authors:** Catharine Pearce, Adele Crapnell, Eihab O. Bedawi, Najib M. Rahman, John P. Corcoran

**Affiliations:** 1 Interventional Pulmonology Service, Department of Respiratory Medicine, University Hospitals Plymouth NHS Trust, Plymouth PL6 8DH, UKadelecrapnell@nhs.net (A.C.); 2 Department of Respiratory Medicine, Sheffield Teaching Hospitals NHS Foundation Trust, Sheffield S10 2JF, UK; eihab.bedawi3@nhs.net; 3 University of Oxford Respiratory Trials Unit, Churchill Hospital, Oxford OX3 7LE, UK; 4 NIHR Oxford Biomedical Research Centre, University of Oxford, Oxford OX3 7JX, UK; 5 Academic Department of Respiratory Medicine, Royal Devon and Exeter Hospital, Exeter EX2 5DW, UK

**Keywords:** pleural infection, empyema, parapneumonic effusion, intrapleural fibrinolytics

## Abstract

Pleural infection represents a significant and ongoing challenge for patients, clinicians, and healthcare providers given the morbidity and mortality associated with this condition. Whilst our understanding of how pleural infection develops and how it should be treated has improved considerably over the past couple of decades, this has yet to translate into a meaningful positive impact on key outcomes. Making the diagnosis of pleural infection is not always straightforward, and the long-standing belief that it always occurs as a complication of lung parenchymal infection is being increasingly recognised as incorrect. Identifying the causative organism(s) is equally uncertain, with almost half of cases of pleural infection proving to be culture negative using traditional methods. Whilst we are now able to determine which patients are more likely to have a poor outcome from their pleural infection at the time of diagnosis, how this should affect their treatment pathway—including the role of more invasive strategies such as surgery or intrapleural enzyme therapy—is not yet known. This review article aims to summarise the existing evidence base and best clinical practice for the non-specialist, whilst highlighting recent research which has or will change the way we manage pleural infection, as well as those areas where further studies are still needed.

## 1. Introduction

Pleural infection continues to present many challenges to clinicians and, by extension, patients diagnosed with and treated for this condition. Despite medical and scientific advances in our understanding of the underlying aetiology and pathogenesis, alongside a broader range of therapeutic adjuncts, this is yet to have a significant impact upon the morbidity and mortality associated with this condition. The worldwide incidence of pleural infection continues to increase, and the median length of hospital stay remains between 10 and 14 days despite apparent improvements in clinical knowledge and treatment strategies. Pleural infection continues to have the overall highest average costs per case of any acute respiratory presentation and a 1-year mortality reported as being between 10 and 20%, rising to 35% in more vulnerable populations such as the immunocompromised and the elderly [1,2,3].

## 2. Diagnosis

Pleural infection is best defined as the entry to and subsequent replication of bacteria in the pleural space. This encompasses a spectrum of disease, including complicated parapneumonic effusion (CPPE) and empyema, and recognises primary pleural infection in its own right [4]. The diagnosis of pleural infection can be challenging and relies first and foremost on the awareness of the assessing clinician. The clinical presentation has two predominant phenotypes. Young individuals with few comorbidities often present with an acute febrile illness associated with pleuritic chest pain, breathlessness, and cough with or without sputum production. More vulnerable individuals, such as those who are frail and co-morbid, or immunocompromised, frequently have an insidious presentation with non-specific symptoms such as anorexia, malaise, and weight loss that can lead to alternative diagnoses being pursued before pleural infection is considered and identified. The failure of any individual to respond to appropriate antibiotic therapy for what is presumed to be a ‘simple’ pneumonia should prompt investigation for an associated pleural effusion. Delayed recognition and management of pleural infection inevitably have a negative impact on morbidity and mortality [5,6].

### 2.1. Imaging Studies

Plain chest radiography is the first-line investigation in all acute respiratory presentations and can be useful in the initial detection of pleural effusion, consolidation, or additional lung parenchymal pathology. Unfortunately, the sensitivity is poor and around 10% of significant parapneumonic effusions may be missed on a chest radiograph, particularly when there is a concurrent basal pneumonia [7]. Whilst pleural infection has previously been considered as a direct sequela to pneumonia or other lung parenchymal infection, radiological features of pneumonia are absent in around a third of cases of pleural infection [8].

The use of thoracic ultrasound (TUS) is becoming increasingly common and readily available to clinicians at the patient’s bedside. In comparison to chest radiographs, TUS has increased sensitivity (94.54 vs. 67.68%) and specificity (97.88 vs. 85.30%) in the identification of pleural effusions [9]. Pleural effusions are present in anywhere between 20 and 57% of cases of pneumonia, yet only 5–7% of these are subsequently implicated in the development of pleural infection [10,11]. Knowing when a diagnostic and/or therapeutic pleural intervention is indicated once an effusion is identified can be challenging, and using TUS to characterise fluid with features such as echogenicity and septations relies on the skills and expertise of the individual operator to elicit them [12]. There are, however, no robust data to suggest that TUS can either diagnose or exclude pleural infection without the need for more invasive intervention, whilst efforts to risk stratify patients with pleural infection using sonographic appearances have also proven unsuccessful. Whilst the presence of septations has intuitively been considered as one sonographic marker that might impact outcome, previous work has shown that their presence and/or severity do not appear to correlate with key clinical outcome measures, including the need for surgery at 3 months, length of hospital stay, or readmission at 12 months [13].

Recognising the limitations of certain imaging modalities in detecting pleural effusions and lack of data in this area of practice, a single-centre feasibility study explored the routine use of early front-door TUS in patients diagnosed with and treated for pneumonia. A total of 41 individuals with either chest X-ray (CXR) or computed tomography (CT) changes in keeping with consolidation proceeded to have a TUS performed within 24 h of their initial diagnosis. A total of 26 (63%) individuals had evidence of a pleural effusion on TUS, with 19 cases being detected on TUS alone. Additionally, 2 cases of pleural infection were subsequently confirmed following diagnostic thoracocentesis [14]. A larger prospective evaluation is required to assess the significance of these findings, recognising this more agile and proactive approach may detect suspected cases of pleural infection much sooner and minimise potential treatment delays.

The use of venous phase contrast-enhanced CT is recommended in all individuals with ongoing pleural sepsis beyond 48 h of chest tube drainage and optimal medical management, and potentially sooner than this in more complex or concerning cases. This modality allows the clinician to review pleura, parenchyma, mediastinal pathologies, and chest tube position as part of a comprehensive radiologic assessment to guide ongoing management. Pleural contrast enhancement, microbubbles within the pleural collection, extra-pleural fat attenuation, and fluid volumes > 400 mL have all been retrospectively identified as features highly suggestive of pleural infection. The CT score created with these parameters was reported as being 84% sensitive and 75% specific for the diagnosis of pleural infection [15], but requires prospective validation alongside a meaningful evaluation of how the use of this score might positively impact on clinical decision making and key outcomes given the implications for both resource provision and the routine use of ionising radiation in all patients with suspected pleural infection otherwise.

### 2.2. Pleural Fluid Analysis

The diagnosis of pleural infection is confirmed following diagnostic thoracocentesis. The aspiration of pus confirms a macroscopic diagnosis of pleural infection (empyema), whilst a microscopic diagnosis can be made in the presence of either positive pleural fluid cultures and/or the identification of microorganisms on Gram stain. However, incurring a further delay whilst waiting for the microbiological results in the context of high clinical suspicion for pleural infection is undesirable. Consequently, respiratory physicians use point-of-care pleural fluid biochemistry, namely, pH, glucose, and lactate dehydrogenase (LDH), to establish a diagnosis in a timelier manner [4].

Pleural fluid pH is the biochemical parameter with the highest diagnostic accuracy in parapneumonic effusions [16,17]. The cut-off for a pH ≤ 7.2 warranting chest tube insertion in CPPE is used in both American College of Chest Physicians [18] and more recent British Thoracic Society guidelines [19], and based on the results of a meta-analysis of pleural fluid analysis studies in patients with parapneumonic effusion [16], as well as a prospective study of 238 patients with parapneumonic effusion that identified the highest pH in the CPPE group to be 7.38, whilst only 14/159 patients with uncomplicated parapneumonic effusion had pH ≤ 7.2 [17]. The interpretation of pleural fluid pH should always be applied to the presenting clinical context; low pH pleural effusions can also be related to other common conditions such as rheumatoid or malignant effusions. Furthermore, pleural fluid pH samples should not be contaminated with local anaesthetic or heparin, as these can artificially lower pleural fluid pH and confound the diagnosis [20]. If there is any diagnostic doubt or pleural fluid pH is unavailable, a pleural fluid glucose of <3.3 mmol/L can be used as a surrogate marker whilst, once again, taking clinical context into account and considering alternative causes [19,20]. The independent use of pleural fluid LDH is a poor predictor of CPPE in isolation, but high levels in the thousands can offer additional weight in the correct clinical context and where findings with either pleural fluid pH or glucose are felt to be borderline or inconclusive [19,20].

### 2.3. Baseline Predictors of Outcome

Until recently, it has not been possible to reliably identify individuals with pleural infection who are at higher risk from their condition and more likely to have poor clinical outcomes. The RAPID score (Table 1) is the first prospectively validated risk stratification score in pleural infection to achieve this, using five parameters (renal function (serum urea), age, purulence of pleural fluid, infection source (community- or hospital-acquired), and dietary status (serum albumin)) routinely available at baseline presentation to predict outcome (Table 1) [2]. Low- (RAPID 0–2), medium- (RAPID 3–4), and high-risk (RAPID 5–7) groups were associated with a 3-month mortality of 3%, 9%, and 31%, and a median length of hospital stay of 7 days, 10 days, and 15 days, respectively [3]. Whilst a validated risk stratification tool has intuitive benefits and allows clinicians to have more informed discussions with their patients about the likely direction of travel, the role it might play in clinical decision making is yet to be clearly defined. A recent feasibility study assessing the impact of more aggressive management strategies for pleural infection—whether surgical (video-assisted thoracoscopic drainage) or medical (intrapleural enzyme therapy (IET))—appeared to show an improvement in length of hospital stay with these interventions when compared to standard care [21], and their targeted use in high-risk cases identified using the RAPID score may be one way in which this could inform clinical decision making in time [19].

## 3. Microbiology

The mainstay of treatment in pleural infection is the early initiation of appropriate antibiotics alongside the timely and effective drainage of infected pleural fluid. Knowledge of the predominant organism is fundamental to achieving adequate antibiotic coverage. Unfortunately, the complex microbiome of pleural infection is not yet fully understood and is consequently a potential barrier to our progress in managing this condition, both diagnostically and therapeutically [22,23].

Although pleural effusions may develop in up to half of all cases of pneumonia, an evolution that is associated with a significant increase in morbidity and mortality [24], it is increasingly accepted that pleural infection represents a distinct clinical entity. This is evident not only from the third of cases of pleural infection without any demonstrable pneumonia on imaging [8] but also the variation in microbiology, which means several common pathogens associated with atypical pneumonias are almost completely absent in pleural infection [22,25,26]. It is now widely recognised that pleural infection may evolve as a consequence of multiple clinically discrete processes that go beyond it being a straightforward complication of pneumonia, with examples, including haematological dissemination and direct seeding of the pleural space; micro-aspiration of oral commensals; and trans-diaphragmatic spread of microorganisms more ordinarily found in the gastrointestinal tract [4].

The geographical variation in pathogens responsible for pleural infection is now also widely recognised [22]. The subtropics report a higher incidence of Gram-negative bacteria in comparison to the tropics and temperate regions where a higher incidence of Gram-positive bacteria is reported. The most common pathogen both in the subtropics and worldwide is *Staphylococcus aureus*; whilst the viridans streptococci and *Streptococcus pneumoniae* are more commonly identified in temperate regions and the tropics. Whilst these patterns are not fully understood, health behaviours are expected to have a major influence on this. Furthermore, there are distinct differences in pathogens dependent on the setting of infection (hospital- vs. community-acquired) (Table 2). Gram-positive aerobes are dominant in community-acquired infection, whereas Gram-negative aerobes and more resistant organisms are seen in hospital-acquired infection. Methicillin-resistant *Staphylococcus aureus* (MRSA) is more commonly detected in hospital-acquired pleural infection and appears to be identified with increasing frequency over time, particularly in the subtropics. This highlights the importance of selecting empirical antibiotics in conjunction with local policy and known resistance patterns within that region [19,22].

### 3.1. Improving Diagnostic Yield

Conventional pleural fluid cultures are positive in just 58% of cases, meaning a significant minority of patients are dependent on empiric therapy with a potential negative impact on morbidity and mortality as a result [22,25]. The low diagnostic yield of pleural fluid culture is hypothesised to be due to one or more factors, including low bacterial concentrations within pleural fluid, the use of broad-spectrum antimicrobial therapy prior to sampling, and/or the unfavourable hypoxic pleural environment nurturing pathogens that are subsequently difficult to culture in vitro. A straightforward intervention shown to improve diagnostic yield in a prospective study [27] is the inoculation of blood culture bottles with pleural fluid as a transport and culture medium, alongside more traditional plain bottle culture. This increased diagnostic yield by up to 20% (*p* < 0.001) and has led to a widely accepted change in practice and guidelines [19,28], whereby pleural fluid is sent for culture in both universal containers and blood culture bottles.

Despite the measures described above, a persistently low microbiological yield from pleural fluid has led clinicians and researchers to consider whether the correct site is being targeted. A feasibility study [29] looked at whether TUS-guided pleural biopsy immediately prior to chest drain insertion in patients with an established diagnosis of pleural infection might improve diagnostic accuracy. Although a small study with just 20 participants, this approach increased microbiological yield from conventional methods by up to 25%, with no adverse outcomes reported and despite the majority of patients included already being established on broad-spectrum antibiotics. These findings may imply that bacterial pathogens prefer the highly vascularised parietal pleural surface rather than suspension within the pleural fluid itself, a hypothesis that seems instinctively logical. A larger prospective study is necessary to identify the true significance of this approach, including whether it can impact relevant clinical outcomes, including antibiotic selection and/or duration.

Further efforts to address shortcomings in our knowledge of the pleural infection microbiome has seen an increasing interest in the use of next generation sequencing, with a pioneering early study [30] analysing pleural fluid samples from the MIST-1 cohort [31]. Of 434 pleural fluid samples collected during the original MIST-1 study, 58% were culture positive by standard measures (Gram-stain and culture), with 35% being single aerobic, 9% single anaerobic, 12% polymicrobial, and 2% Gram-stain positive only. In total, 77 subjects had received antibiotics prior to pleural fluid sampling, with 61% of these cases being culture negative. A total of 404 fluid samples were available for detailed molecular testing, identifying potentially pathogenic bacteria in an additional 16% of cases, which had originally been labelled as culture negative. The remaining 26% of the original 434 cases had undetermined microbiology. A further subset analysis demonstrated a clear microbiological distinction between pleural infection and pneumonia, with Streptococcal infection being the dominant community-acquired pathogen (52%), whilst hospital-acquired infection *Staphylococcus aureus* and enterococci were the dominant isolates, with 25% of the former group being methicillin-resistant. These differences in the microbiome have been subsequently proposed as a major contributing factor to the increased mortality and morbidity observed in hospital-acquired pleural infection [1,2,30].

The more recent TORPIDS study [23] built on this preceding work by using 16S rRNA next-generation sequencing to analyse pleural fluid samples collected as part of the PILOT study [3], demonstrating 79% of samples to be polymicrobial and 33.5% to have anaerobic predominance. Within this cohort, key microbiological patterns were identified in mono-microbial versus polymicrobial infection, as well as community- versus hospital-acquired infection, and the interplay between these two distinctions. Infections with *Staphylococcus aureus* and Enterobacteriaceae, where resistance to widely used empiric antibiotics is well documented, were clearly associated with higher morbidity and mortality. Whilst these newer molecular sequencing techniques are less likely to be impacted by prior antibiotic use, thereby overcoming some of the challenges encountered with conventional culture methods, they are more likely to identify polymicrobial samples; establishing which isolate is of the greatest clinical significance may, therefore, be challenging and further research is required around this area of practice. This may, however, allow for further refinement of risk stratification models based upon microbiology if these tools are more widely available and able to provide results early on in the course of a patient’s illness.

### 3.2. Fungal Pleural Infection

Fungal involvement is fortunately rare, being seen in less than 1% of cases of pleural infection, but represents a serious subset of the condition with an in-hospital mortality of up to 38% and most commonly occurring in the immunocompromised patient population [32,33]. In the vast majority of healthy and immune competent individuals, however, a positive fungal culture from pleural fluid is most likely to be a contaminant; confirmed fungal pleural infection should prompt investigation for HIV and other potential causes of immunodeficiency. Treatment, otherwise, follows standard guidelines with the use of appropriate antifungals based on culture findings and local microbiology advice.

### 3.3. Tuberculous Pleuritis

Tuberculosis (TB) remains a major global health burden associated with significant morbidity and mortality. Whilst a detailed review of its diagnosis and management [34] lies outside the scope of this particular article, clinicians should be aware of and alert to the possible diagnosis of TB pleuritis in patients with a history of exposure or other underlying risk factors such as immunocompromise, and/or where the infection is endemic within the local population. As with conventional bacterial pleural infection, maintaining a high level of vigilance is key as both the clinical and radiologic features on presentation can be non-specific and overlap with other potential diagnoses including malignant pleural disease. As TB pleuritis is typically paucibacillary, the sensitivity of conventional tests such as microscopy for acid-fast bacilli and TB culture from pleural fluid is low; nucleic acid amplification tests, whilst useful, underperform for the same reasons. Obtaining pleural tissue biopsies greatly improves diagnostic yield either through the identification of caseating granuloma on histology and/or positive culture but may not always be technically or practically possible. The diagnosis is, therefore, often dependent on clinical suspicion once other potential causes for the patient’s presentation have been ruled out; alongside typical laboratory findings including lymphocytic cytology and raised adenosine deaminase levels in pleural fluid, and evidence of clinical and radiologic improvement over time with an empiric trial of anti-tuberculous treatment according to local guidelines.

## 4. Treatment

### 4.1. Antibiotic Selection

The initial treatment of suspected pleural infection should commence prior to the availability of any culture results, as any delay can be associated with increased morbidity and mortality. Whether community- or hospital-acquired, pleural infection is predominantly polymicrobial [22,28]. Broad-spectrum empiric antibiotic treatment is, therefore, required in almost all cases to begin with and should include anaerobic cover (Table 3), with the precise choice of agent(s) informed by both local guidelines and knowledge of locoregional microbiological prevalence. Early discussion with local microbiology services is encouraged. In hospital-acquired infection, consideration should be given to cover for Gram-negative organisms and MRSA [22,25]. Should pleural fluid or blood culture results be available either at the outset or later in the course of treatment, this may allow a more focused antibiotic regimen to be utilised. This is particularly true in pneumococcal disease, as this is usually a monomicrobial infection [23].

The duration of antibiotic treatment for pleural infection in adults is usually anywhere between 2 and 6 weeks depending on a combination of clinical and biochemical response alongside clinician confidence [19,28]. This is largely based on expert opinion, with there being limited published data to inform the duration of antibiotic therapy in the context of pleural infection. An open-label randomised controlled trial of 50 patients aimed to assess whether shorter antibiotic duration resulted in more treatment failures when compared to standard longer courses [35]. Adult patients with stable, medically treated pleural infection were randomised to either a short course (14–21 days) or long course (28–42 days) of antibiotic therapy. Patients with a baseline RAPID score > 4 (high-risk category) were excluded from this study. In total, 25 patients were recruited to the short course, and 25 patients to the long course, with outcome data available for 24 patients in each study arm. The only statistically significant outcome was the median duration of antibiotic therapy, which, in the short course group, was 20.5 days, and 34.5 days in the long course group (*p* < 0.001). There were no other statistically significant differences, and, notably, there was no difference in the rate of treatment failure between the long- and short-course antibiotic therapy groups. However, long-term outcome data (such as one-year mortality) were not collected. Furthermore, as a single-centre study with a small sample size, outcomes are likely to be insufficiently powered to influence wider change in clinical practice for the time being. A separate study comparing the efficacy of a two-week versus three-week course of co-amoxiclav for patients with CPPE was underpowered and did not reach statistical significance in any of its key outcomes [36]. Further large-scale prospective multicentre studies are needed to better inform decision making in this area of practice.

There are no direct comparative studies between the use of intravenous and oral antibiotics in pleural infection. Small-scale studies in animals and humans have explored antibiotic penetrance of the pleural space, with antibiotics commonly used in the treatment of pleural infection (e.g., amoxicillin, metronidazole, piperacillin–tazobactam, and clindamycin) all reaching levels in the pleural fluid equivalent to that in the bloodstream and above the applicable minimum inhibitory concentrations [37]. Conventional practice continues to begin with intravenous therapy, transitioning to oral therapy based on the physiological and biochemical response to treatment. Home intravenous treatment may be considered via ambulatory or community-based services if there are reasons for oral therapy not being feasible or appropriate.

### 4.2. Medical Management of the Infected Pleural Space

Adequate drainage of the infected fluid and other material from the pleural space, thereby achieving source control, is critical to the successful management of pleural infection. When the pleural fluid obtained at initial diagnostic thoracocentesis is purulent (empyema thoracis), this is macroscopically diagnostic for pleural infection and the responsible clinician(s) should proceed immediately to intercostal chest drain insertion without the requirement for further biochemical analysis. Factors determining those patients with non-purulent pleural fluid who also require intercostal drainage include clinical, radiological (CT and TUS), and biochemical (pleural fluid pH, glucose, and lactate dehydrogenase (LDH)) parameters (Table 4). The key caveat around intercostal chest drain insertion is that there should always be a safe volume of accessible fluid demonstrated on either TUS and/or CT to warrant the intervention; if in doubt, either further imaging and/or a second expert opinion is advisable.

The size of the initial chest drain appears to have no definite effect on either mortality, the requirement for repeat pleural intervention, or duration of hospital stay. A bore size > 14F may, however, cause post-treatment pain [38]. On that basis, the choice of any initial intercostal chest drain for pleural infection is recommended to be ≤14F in recently published guidelines [19,28]. Repeated therapeutic thoracocentesis with drainage to dryness is a potential management strategy in carefully selected cases and under close specialist supervision, with the responsible clinician(s) likely to base the decision to pursue this approach on a range of factors, including baseline performance status, systemic physiological parameters, and the size of the infected collection. Whilst this approach has been described and evaluated in single-centre studies and shown to be well tolerated [39,40,41], there are no robust or validated criteria to facilitate patient selection, nor are there any adequately powered randomised prospective multicentre studies to demonstrate the safety and efficacy of this management strategy. As such, it is not recommended in guidelines for the time being and remains highly restricted on a case-by-case basis under close expert oversight.

### 4.3. Adjuncts to Chest Tube Drainage

A complex pleural effusion, most commonly described as being either septated and/or loculated on imaging studies, reduces the likely success of simple chest tube drainage, and may necessitate a more invasive approach to subsequent management. The progressive organisation of inflammatory pleural fluid develops as a consequence of dysregulation of the fibrinolysis cascade in response to proinflammatory stimuli and is part of the natural history of an infected pleural space with the rate and extent of progression varying between individuals. This process may begin with the development of fibrinous deposition, resulting in pleural adhesions and loculations that become more complex over time and are often associated with the development of a pleural rind [42,43]. Potential biomarkers in pleural fluid have been identified that may predict the predilection of an effusion, whether infected or not, to become more complex over time; these are not currently used to diagnose pleural infection, however, and further research is required to investigate their diagnostic accuracy and allow prospective validation of their use.

Plasminogen activator inhibitor-1 (PAI-1) is the primary inhibitor of tissue- and urinary-type plasminogen activators, enzymes responsible for the degradation of fibrin, and has been established to be raised in infected pleural fluid [13,43,44]. Analysis of pleural fluid samples collected as part of a prospective, observational pleural infection study [3] found PAI-1 levels to be associated with pleural septation presence and severity, increased length of hospital stay, and increased 12-month mortality [13]. These findings are, however, limited by the pleural fluid samples available for analysis being taken at the time of diagnosis with pleural infection; as such, it could not explore whether sustained elevations in PAI-1 would lead to increased pleural septations and reduce the likelihood of success with chest drain insertion alone. Baseline PAI-1 levels may additionally not reflect those in later infection stages due to individual case variability in pleural fluid microbiology, co-morbidities, and/or response to antibiotics over time [13,43].

Soluble urokinase plasminogen activator receptor (suPAR) is a protein present in various biological fluids, including pleural effusions [45], with elevated suPAR levels having been demonstrated in inflammatory pleural conditions, including infected and malignant effusions [46]. A single-centre prospective cohort study found suPAR pleural fluid levels in parapneumonic effusions to be significantly higher in loculated compared to non-loculated effusions and, consequently, predictive of the need for chest drain insertion. Furthermore, when predicting the need for either intrapleural enzyme therapy or thoracic surgery referral, pleural suPAR levels appeared to be superior to conventional pleural fluid biomarkers (pH, glucose, and lactate dehydrogenase) [47]. The precise utility of pleural suPAR in complex septated or loculated effusions may be challenging, however, due to its presence in effusions of both malignant and infective aetiologies; this warrants further study to better define its place as a diagnostic and/or prognostic tool in pleural disease.

Whilst small-bore chest drains are recommended as the first-line intervention for an infected pleural collection [19,38], they are very likely to become blocked without appropriate measures to maintain their patency [48]. The ideal volume and frequency of chest drain flushing has not been established, but a retrospective study has shown that six-hourly 20 mL 0.9% saline flush administration appears sufficient to significantly reduce the frequency of small-bore chest drain occlusion [49]. Taking this approach a step further and extrapolating from post-operative management described in prior case series [50,51], a single-centre randomised controlled pilot study (*n* = 35 participants) compared intrapleural saline irrigation (250 mL three times daily for up to three days) with standard clinical care (30 mL saline flushes three times daily) [52]. In the saline irrigation group, there was a significantly greater reduction in pleural collection volume on CT and significantly fewer patients referred for surgery; no difference was observed in length of hospital admission, improvement in inflammatory markers, time to resolution of fever, or adverse events between the groups. The authors concluded that further large-scale multicentre studies were needed to better evaluate this approach; however, the available data were sufficient to allow intrapleural saline irrigation to be included in recent guidelines as a potential adjunct to chest tube drainage where other salvage strategies (for example, thoracic surgery or intrapleural enzyme therapy) are either unavailable or unsuitable [19].

### 4.4. Intrapleural Enzyme Therapy (IET)

The role of intrapleural enzyme therapy (IET) for pleural empyema and CPPE has been studied since the 1940s [53], but it is only in the past two decades that large-scale multicentre trials have been undertaken to inform the evidence base for its continued use. In the MIST1 randomised controlled trial, intrapleural streptokinase was compared to placebo in 454 adult patients with pleural infection [31]. There was no significant difference in mortality, surgical referral, radiographic outcome, or length of hospital stay between the groups; there was, however, a non-significantly increased rate of adverse events (*p* = 0.08) with the use of streptokinase. A subsequent Cochrane review of intrapleural fibrinolytics (streptokinase, alteplase, or urokinase) versus placebo in empyema or CPPE found no difference in mortality between the fibrinolytic and placebo treatment groups, and whilst a reduction in the requirement for surgery and overall treatment failure with the use of fibrinolytics this benefit was lost once studies felt to be at a high risk of bias were excluded from the analysis [54].

In the context of evolving pleural infection, the accumulation of extracellular DNA and bacterial components is thought to contribute to increasing pleural fluid viscosity and the formation of biofilms over time [55,56,57]. In vitro studies not only demonstrated the importance of disrupting septations with fibrinolytic agents but also the role of deoxyribonuclease (DNase) in altering the viscosity of the fluid and biofilm formation as a means of improving drainage [58,59]. The MIST2 randomised controlled trial subsequently explored the effect of a directly acting fibrinolytic, tissue plasminogen activator (tPA), in combination with human DNase [60]. In total, 210 adult patients with pleural infection were randomised to one of four study arms: double placebo, tPA and placebo, DNase and placebo, or intrapleural tPA and DNase. When compared to placebo, the combination of intrapleural tPA and DNase led to statistically significant reductions in surgical referral and length of hospital stay, as well as a significant improvement in the drainage of the infected collection as measured on chest radiograph. By contrast, neither single-agent tPA nor single-agent DNase improved these same clinical outcomes in comparison to placebo [60].

Based on these trial data, combination IET using tPA and DNase together is recommended when initial chest drainage has ceased and a clinically concerning pleural collection persists [19,28]. The standard regimen advised in guidelines [19,28] remains the same as that used in the MIST2 trial [60]: 10 mg tPA twice daily and 5 mg DNase twice daily for three days. There is a small but widely recognised increase in the risk of bleeding with the use of combination IET, particularly in the context of therapeutic anticoagulation, increased RAPID score, elevated serum urea, and platelets <100 × 10^9^/L [61]. Although the overall risk of bleeding was low (4.1%), patients should specifically consent for this, and, if able, anticoagulation should be withheld prior to the use of IET [19,28,61]. The use of a half dose of tPA (5 mg twice daily) mitigates the risk of bleeding somewhat and should be considered in patients for whom the cessation of therapeutic anticoagulation is not possible [61]. There have been small prospective studies demonstrating the safety and clinical efficacy of dose-reduced tPA [62,63], but these have been limited by a lack of comparative study arms using a conventional dose of tPA or no IET at all; as such, optimising and/or personalising the dose of IET needed to achieve the required outcome remains a subject for ongoing research.

### 4.5. Surgical Management of the Infected Pleural Space

Although optimised medical management as described above will be successful in most patients with pleural infection, a significant minority—in the region of 20% in prospective multicentre studies enrolling all-comers with pleural infection [3,31]—will fail to improve. This may be due to a failure to respond to antimicrobial treatment, a persistent effusion that has failed to clear despite chest tube insertion and additional adjuncts, and/or ongoing sepsis. The chronicity of pleural infection and delays in either treatment or presentation to healthcare services may additionally contribute through potential progression in the complexity of the space, with fluid loculation and the evolution of pleural thickening with rind formation and consequent trapped lung [5,6].

There is, at the present time, no widely accepted consensus on the role and timing of surgical intervention for pleural infection. As such, recently published physician-led guidelines [19,28] state that surgical treatment, including medical thoracoscopy, should not be considered as the initial treatment modality for pleural infection. Other guidelines produced by surgical societies [64,65] support a more interventional approach whereby patients with complex infected pleural collections, and who are deemed fit for operative management, should be considered for early surgical treatment.

Modern surgical intervention for pleural infection usually entails an endoscopic approach via video-associated thoracoscopic surgery (VATS), and less commonly an open approach with thoracotomy. Compared to thoracotomy, VATS has demonstrated more favourable outcomes with regard to length of hospital stay, morbidity, pain, post-operative air-leak, operative duration, time to return to work, and in areas of patient acceptability, including satisfaction with the wound and the operation overall [66,67,68]. A key consideration in surgical management is selecting a technique that allows debridement for optimal clearance of the infected pleural space, in addition to restoring lung expansion in those patients in whom a pleural rind has resulted in trapped lung. The results of a Society of Thoracic Surgeons study [5] exploring outcomes from surgical decortication for empyema and pleural effusion demonstrated that risk factors for death included age, renal function, COPD, baseline performance status, and time between admission and operative intervention. Decortication represents a higher risk procedure for comorbid individuals and requires careful expert input to make an individualised decision for the patient in whom trapped lung has occurred as a consequence of pleural infection [28,65].

Older patients often have higher mortality associated with pleural infection, likely as a consequence of multimorbidity and reduced physiological reserve [1,69,70]. These same co-morbidities have also represented a traditional barrier to surgery, with case series reporting surgical outcomes in pleural infection having demonstrated a potential bias towards younger patients being offered this treatment more readily [3,4]. A retrospective single centre study of uniportal VATS in stage II pleural empyema (American Association for Thoracic Surgery criteria) demonstrated an increased operative duration, chest tube remaining in situ for longer, and greater length of hospital stay in patients over 70 years of age, when compared to adults under 70 years old [71]. The study authors attributed these findings to the presence of comorbidities in the over 70 age group. No comparison was made, however, between different surgical techniques nor the use of intrapleural enzyme therapy.

The recently published MIST-3 feasibility trial [21] was the first study of its kind to assess the early application of video-assisted thoracoscopic surgery (VATS) or intrapleural enzyme therapy (IET) in adult patients with pleural infection. There was evidence of a potential reduction in the length of hospital admission with surgical treatment, and possible earlier resolution of pain and shorter recovery times with IET. Further research in this area is now vital to determine the optimal initial management approach in pleural infection and identify those cases most likely to benefit from early and more aggressive treatment strategies.

### 4.6. Other Treatment Considerations

Medical thoracoscopy (MT), performed under conscious sedation and local anaesthetic, has been considered as an alternative and less invasive option to traditional surgical methods for the management of pleural infection. There is, however, a lack of robust prospective data to define its place in the treatment of this condition, and, therefore, current international guidelines [19,28] do not recommend its routine use for the management of pleural infection. A meta-analysis of eight studies explored the role of MT for the treatment of pleural infection, either as a first-line intervention or following failure to improve with chest tube drainage alone [72]. MT had a success rate of between 75 and 94% across the studies, defined as either the absence of any need for further surgical intervention and/or radiological and clinical improvement. Three of the studies included in the analysis explored the use of post-thoracoscopy intrapleural fibrinolysis, with only one of these using combination IET (fibrinolytic and DNase). The reported success rate across these studies was between 81.8 and 96.8%, whilst the pooled difference in the success rate between those that received fibrinolytics and those that did not was 9%, favouring fibrinolysis. However, there was significant heterogeneity across the studies included in this analysis, with the timing of any pleural intervention and the precise MT approach used being unclear, in addition to the definition of treatment success differing between the studies.

In patients with non-resolving chronic pleural infection and who are not candidates for surgical treatment, the long-term management approach can be challenging whilst both morbidity and mortality are predictably high. Facilitating source control with, for example, the use of an indwelling pleural catheter has been successfully described in select cases of chronic pleural infection that have failed to resolve completely with optimal medical management [73,74]. Long-term antibiotic suppression therapy may also be considered alongside or separate to a drainage strategy with a view to safely managing the patient outside of an acute inpatient hospital environment.

In the setting of increasingly complex disease and the associated need for case-by-case discussion, it is recommended to seek expert advice. This may be via regional colleagues in the first instance. In the United Kingdom, a national pleural MDT has been recently established [75]. This is a forum of national pleural experts (chest physicians, thoracic surgeons, pathologists, and radiologists), meeting virtually every two months, that gives clinicians an opportunity to access expert and consensus advice on more difficult cases.

## 5. Future Directions

Despite the significant progress that has been made in our understanding of how and why pleural infection develops, and a growing range of approaches to its subsequent management, we still have major gaps in our knowledge that continue to limit the gains that might be made in terms of both the morbidity and mortality associated with this condition. Understanding why parapneumonic effusions develop and then progress to become complicated in some patients but not others is likely to be central to early diagnostic and even preventative strategies; it may be those cases of pleural infection that occur without any apparent prior lung parenchymal involvement [8] that offer us greater insight into the pathophysiology underlying these processes.

Whilst we now have a validated risk stratification score [2,3] that allows clinicians to predict key clinical outcomes at the time of presentation and diagnosis with pleural infection, further research is needed to see if directing management strategies according to RAPID score can have a positive impact on patient care. Understanding whether this risk stratification process can be either enhanced or personalised through the use of imaging or serum biomarkers [13,47], and/or better microbiological definition through early and wider access to novel sequencing techniques [23], merits more detailed consideration in the coming years.

Our approach to the treatment of pleural infection also needs further refining. The role and timing of more invasive and aggressive strategies, whether IET or surgical, remains unclear given the signal towards improved outcomes with their early use [21]—a definitive randomised trial is needed to answer this specific question and ensure the correct patient population has access to these treatments if they do prove to be beneficial. At a more fundamental level, the choice and duration of antibiotic treatment and our ability to personalise this to an individual patient’s needs based on proven markers of clinical, biochemical, and radiologic recovery remains an area of continued uncertainty; yet one that needs critical attention in the face of growing concerns around antimicrobial resistance and appropriate stewardship.

## 6. Conclusions

**Figure 1:**
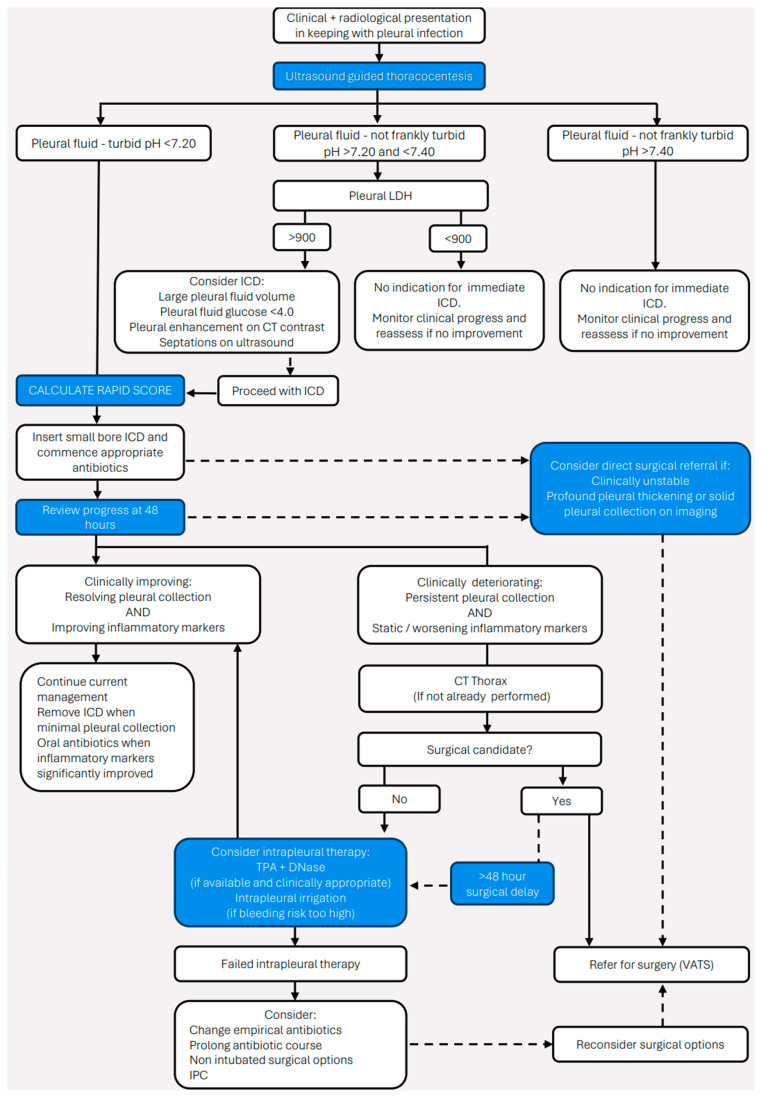
Algorithm for diagnosis and management of pleural infection (changes to prior British Thoracic Society guidance highlighted for information) [19].

Pleural infection represents an ongoing, even growing challenge for patients, clinicians, and healthcare providers alike. Despite the progress that has been made with both diagnostic and therapeutic approaches, underpinned by robust clinical research, there continues to be significant morbidity and mortality that requires a further paradigm shift in how we manage this condition. For now, early recognition and diagnosis, allied to broad spectrum antibiotic treatment and source control through either medical or surgical drainage of the infected pleural space remains central to best clinical practice (Figure 1). However, the ability to reliably identify the most vulnerable patients at the time of diagnosis must now be translated into evidence-based treatment pathways that improve key clinical outcomes, whilst precise and personalised care may be achieved through widening access to new microbiologic tools and predictive biomarkers as these are validated as part of ongoing studies. It is only through a greater understanding of how, why, and when pleural infection develops, alongside what treatment approach to use and when, that we see a meaningful improvement in the individual patient journey and how it ultimately concludes.

## 7. Key Learning Points

- Pleural infection remains a major challenge to clinicians, patients, and healthcare providers, with an increasing incidence and persistently high morbidity and mortality despite improvements in diagnostic and therapeutic approaches.
- Pleural fluid analysis for key biochemical (pH and glucose) markers remains the gold standard for diagnosis to be used alongside conventional culture techniques, although between a third and half of cases will prove to be microbiology negative.
- The management of pleural infection is centred on appropriate antibiotic selection and the adequate clearance of infected material from the pleural space using either medical (aspiration, chest tube drainage with or without intrapleural enzyme therapy) and/or surgical techniques.
- The RAPID score allows the risk stratification of patients with pleural infection at baseline presentation, but there are no robust data that show how this could or should influence clinical decision making for the time being.
- Future research must focus on novel laboratory techniques to improve diagnosis and prognosis in pleural infection, and on personalised management strategies that are tailored according to baseline risk stratification (RAPID) score and/or other clinical characteristics.

## Figures and Tables

**Table 1:** RAPID score for risk stratification of pleural infection in adults—adapted from Rahman et al. [2], doi: 10.1378/chest.13-1558 with permission of the American College of Chest Physicians.

	Parameter	Score
**Renal function (Urea mM)**	<5	0
	5–8	1
	>8	2
**Age (years)**	<50	0
	50–70	1
	>70	2
**Purulence of pleural fluid**	Purulent	0
	Not purulent	1
**Infection source**	Community	0
	Hospital	1
**Dietary Status** **(Albumin g/L)**	>27	0
	<27	1
**RAPID Score**	0–2	Low risk
	3–4	Medium risk
	5–7	High risk

**Table 2:** Common microbiologic isolates from pleural fluid culture in community- and hospital-acquired cases of pleural infection, in decreasing order of frequency [22].

Community Acquired	Hospital Acquired
*Viridans streptococci*	*Staph aureus*
*Pneumococci*	*Enterobacteriaceae*
*Staph aureus*	*Enterococci*
*Enterobacteriaceae*	*Viridans streptococci*
*Klebsiella*	*Pseudomonas*
*Pseudomonas*	*Klebsiella*

**Table 3:** Examples of empiric antibiotic regimes for use in adult cases of pleural infection (assuming normal renal and liver function).

	Community-Acquired	Hospital-Acquired
**1st line**	Co-amoxiclav 1.2 gm IV TDS	Piperacillin + Tazobactam 4.5 gm IV TDS
**1st line (penicillin allergy)**	Levofloxacin 500 mg IV/PO BD Metronidazole 400 mg PO (or 500 mg IV) TDS	
**If MRSA + ve**	Add teicoplanin IV 6 mg/kg with monitoring of levels and dose titration per local protocol thereafter	
**If known or high-risk for ESBL/AmpC colonisation**	Liaise with local microbiology for guidance Consider IV meropenem 1 gm IV TDS initially, de-escalate according to cultures and/or clinical response to treatment	

**Table 4:** Recommendations for decision making around pleural fluid drainage based on initial pH results [19].

Pleural Fluid pH	Likelihood of Pleural Infection	Outcome
≤7.2	High	ICD indicated
>7.2 and <7.4	Intermediate	Consider ICD if LDH > 900, particularly if any of the following additionally present: - Ongoing pyrexia - Large volume effusion - Low pleural fluid glucose < 4.0 mmoV/L - Pleural enhancement on contrast CT - Septations present on ultrasound
≥7.4	Low	No immediate indication for ICD

## Abbreviations

CPPE	complicated parapneumonic effusion
TUS	thoracic ultrasound
CT	computed tomography
CXR	chest X-ray
LDH	lactate dehydrogenase
IET	intrapleural enzyme therapy
MRSA	methicillin-resistant *Staphylococcus aureus*
PAI-1	plasminogen activator inhibitor-1
suPAR	soluble urokinase plasminogen activator receptor
DNase	deoxyribonuclease
tPA	tissue plasminogen activator
VATS	video-assisted thoracoscopic surgery
COPD	chronic obstructive pulmonary disease
MT	medical thoracoscopy
MDT	multidisciplinary team
